# Perceptions and Attitudes Toward Mobile Health in Development of an Exclusive Breastfeeding Tool: Focus Group Study With Caregivers and Health Promoters in the Dominican Republic

**DOI:** 10.2196/20312

**Published:** 2020-08-21

**Authors:** Clarisse G Casilang, Samantha Stonbraker, Ingrid Japa, Mina Halpern, Luz Messina, Andrew P Steenhoff, Elizabeth D Lowenthal, Linda Fleisher

**Affiliations:** 1 Department of Pediatrics Global Health Center The Children's Hospital of Philadelphia Philadelphia, PA United States; 2 Centro de Salud Divina Providencia Consuelo Dominican Republic; 3 Clínica de Familia La Romana La Romana Dominican Republic; 4 Children's Hospital of Orange County Orange, CA United States; 5 School of Nursing Columbia University New York, NY United States; 6 Fox Chase Cancer Center Philadelphia, PA United States

**Keywords:** global health, breast feeding, mHealth, mobile phone

## Abstract

**Background:**

Despite growing interest in the use of technology to improve health outcomes in low- and middle-income countries (LMICs), local attitudes toward mobile health (mHealth) use in these settings are minimally understood. This is especially true in the Dominican Republic, where mHealth interventions are starting to emerge. This information is critical for developing effective mHealth interventions to address public health issues, such as low exclusive breastfeeding (EBF) rates, which can lead to poor outcomes. With an EBF rate of 5% in the first 6 months of life, the Dominican Republic has one of the lowest EBF rates worldwide.

**Objective:**

This study aims to describe the current use of information and communication technology (ICT) and to analyze the attitudes and perceptions related to using mHealth interventions among caregivers of children aged ≤5 years and health promoters in the Dominican Republic. Findings can inform mHealth strategies aimed at improving EBF in this, and other, LMICs.

**Methods:**

Participants were recruited from 3 outpatient sites: the Niños Primeros en Salud program at Centro de Salud Divina Providencia in Consuelo (rural setting) and Clínica de Familia La Romana and its program Módulo de Adolescentes Materno Infantil in La Romana (urban setting). Focus groups were conducted with caregivers and community health promoters to identify the use, attitudes, perceptions, and acceptability of mHealth as well as barriers to EBF. Discussions were conducted in Spanish, guided by semistructured interview guides. All sessions were audio-recorded and later transcribed. Thematic content analysis was conducted in Spanish by two bilingual researchers and was structured around a hybrid behavioral theory framework to identify salient themes.

**Results:**

All participants (N=35) reported having a mobile phone, and 29 (83%) participants had a smartphone. Sources for obtaining health information included the internet, physicians and clinic, family and friends, health promoters, and television. Barriers to mHealth use included the cost of internet service, privacy concerns, and perceived credibility of information sources. Participants indicated the desire for, and willingness to use, an mHealth intervention to support breastfeeding. The desired features of a possible mHealth intervention included offering diverse methods of information delivery such as images and video content, text messages, and person-to-person interaction as well as notifications for appointments, vaccines, and feeding schedules. Other important considerations were internet-free access and content that included maternal and child health self-management topics beyond breastfeeding.

**Conclusions:**

There is a high level of acceptance of ICT tools for breastfeeding promotion among caregivers in urban and rural areas of the Dominican Republic. As mHealth tools can contribute to increased breastfeeding self-efficacy, identifying desirable features of such a tool is necessary to create an effective intervention. Participants wanted to receive trusted and reliable information through various formats and were interested in information beyond breastfeeding.

## Introduction

### Background

eHealth resources hold promise for advancing child health in low- and middle-income countries (LMICs) where information and communication technologies (ICT) are ubiquitous [1]. According to the United Nations Development Program, ICT are a set of “goods, applications or services that are used to distribute and exchange information,” including radio, television, telephone, computers, mobile phones, and the internet [2]. eHealth is the use of ICT in “support of health and health-related fields, including health-care services, health surveillance, health literature, health education, knowledge and research” [3]. Within eHealth, mobile health (mHealth) explores how “mobile technologies can be best used to enhance access to health services and information and to improve the way health professionals deliver health-related services to the general public” [4]. Understanding caregiver perceptions and attitudes toward using mobile phones for health-related purposes is a critical step in developing mHealth interventions that aim to improve child health outcomes internationally, including infant mortality rates [4,5].

Globally, approximately 45% of all infant deaths under 5 years of age are linked to nutrition-related factors [6]. Early initiation of breastfeeding and effective exclusive breastfeeding (EBF) can significantly reduce infant mortality due to common childhood illnesses such as diarrhea or pneumonia [7,8]. According to data from the World Bank, the Dominican Republic has an EBF rate of infants under 6 months of age at only 5% (2014), which is one of the lowest rates worldwide [9]. The Dominican Republic also has an under-five mortality rate of 29 deaths per 1000 live births (2018), considerably higher than the median of 16 deaths per 1000 live births in Latin America and the Caribbean [10]. To address this significant child health issue, breastfeeding promotion interventions emphasizing early initiation and EBF until 6 months of age are critical. These must be effectively designed and utilized in settings such as the Dominican Republic and other LMICs where challenges persist in providing high-quality, easily accessible EBF care and support [11,12].

Although mHealth interventions have been shown to alleviate barriers and enhance access to care in LMICs, a strategic approach in their development is needed to implement effective mHealth on a larger scale and to study how technology can improve health outcomes [4,13-18]. Recognizing that the field of mHealth is rapidly transforming the delivery of health services around the world, the World Health Organization launched an initiative dedicated to the study of eHealth in May 2005 [19]. The United Nations International Children’s Emergency Fund has officially identified “promoting the use of new technologies to more efficiently and effectively serve children, especially the most disadvantaged” as part of its Strategic Plan for 2018-2021 [20]. The literature on mHealth in LMICs has examined the use of technology in health care delivery, health systems development, disease surveillance, and implementation of mHealth-based policies [21-25]. However, caregiver perceptions and attitudes toward using mHealth in a global health setting are poorly understood [26].

### Objectives

The specific objectives of this study were to describe the current use of ICT and analyze the attitudes and perceptions toward mHealth among caregivers of children younger than 5 years in the Dominican Republic. Although previous studies [27-30] have also utilized a stakeholder-informed process to guide the development of an mHealth intervention to promote EBF, this study was designed using a combined behavioral theory model. Findings will inform future mHealth interventions that aim to improve EBF rates in the Dominican Republic and other LMICs.

## Methods

### Institutional Review Board Review

This study was reviewed and granted exemption by the Institutional Review Board at the Children’s Hospital of Philadelphia, which determined that this study met the exemption criteria per 45 CFR 46.104(d) 2. The Comité Nacional de Bioética en Salud in Santo Domingo, Dominican Republic, agreed that the study was exempt from full Institutional Review Board review. All study procedures were conducted in accordance with the ethical standards of the Helsinki Declaration of the World Medical Association.

### Study Sites

The study was conducted at 3 outpatient clinical sites in the Dominican Republic: the Niños Primeros en Salud (NPS) program at Centro de Salud Divina Providencia in Consuelo and Clínica de Familia La Romana (CFLR) and its Módulo de Adolescentes Materno Infantil (MAMI) program, both in La Romana. These sites were purposefully selected to provide a diverse selection of rural and urban mothers and caregivers of children aged ≤5 years to allow for a richer understanding of varied attitudes and perceptions toward mHealth and experiences with EBF. Each site has a set of distinguishing characteristics. In brief, NPS, CFLR, and MAMI fundamentally aim to address factors that influence child nutrition, such as household food insecurity, chronic illness (specifically HIV), and young maternal age, respectively [31]. NPS provides primary outpatient care to children under the age of 5 years living in some of the poorest neighborhoods, also called *barrios*, in a rural community. CFLR is a large health center that provides primary care services and specializes in HIV care and prevention in the Southwest region of the country. MAMI is a satellite clinic of CFLR that provides prenatal and reproductive health care to adolescents and primary care to children of adolescent mothers up to 1 year of age.

### Study Population

Consuelo is a rural municipality of the San Pedro de Macorís province with 30,000 inhabitants [32]. The main industry of the region is agriculture, especially sugar cane production. Many *barrios* in Consuelo lack basic amenities such as indoor plumbing and electricity. The catchment area of La Romana province has a population of 276,000 inhabitants, including 80,000 women aged between 15 to 50 years [33]. An estimated 4000 female commercial sex workers are based in La Romana [33]. In addition to the urban city of La Romana, many sugarcane workers and their families live in *bateyes*, which are underserved sugarcane plantation communities. *Batey* residents include Haitian permanent residents, seasonal migrant workers, and indigent Dominicans [33].

Participants in focus group discussions (FGDs) were stratified into groups of caregivers or community health promoters to encourage comfort and candor, as caregivers may have felt uncomfortable sharing information in front of the promoters. For this study, a caregiver was defined as the mother or female guardian of a child aged ≤5 years receiving care at one of the study sites. Traditionally, health promoters are caregivers selected from within their community to serve as trusted advocates, health educators, and health system navigators for other families in their neighborhood or the surrounding community. CFLR and MAMI health promoters are full-time paid employees, whereas NPS health promoters receive a small stipend for several hours served monthly. As they are considered *experienced parent leaders* in their community, health promoters have a unique perspective on how mHealth can be used to carry out their daily activities.

### Eligibility Criteria

The criteria used for participants to be considered eligible to enroll in the study were as follows:

1. Mother or female guardian of a child aged ≤5 years or female health promoter of NPS, CFLR, or MAMI.
2. For caregiver participants, the child is a patient receiving care at NPS, CFLR, or MAMI.
3. Speaks and understands Spanish.

Due to the sensitive nature of breastfeeding, the lead investigators chose to have female gender as inclusion criteria, as well as a female focus group facilitator. It was inferred that mixed-gender groups might inhibit women from candidly discussing their experiences with, and barriers to, EBF.

### Sample Size

The sample size was established based on the study team’s experiences in group design sessions as well as recommendations for sample sizes in qualitative research [34-36]. Several sources have suggested that well-designed focus groups consist of 6 to 12 participants depending on subject and time limits [37,38]. The rationale for this range stems from the goal that enough participants must be included in the groups for a breadth of information to be captured; however, groups should not be so large that each participant does not have a chance to contribute. Moreover, the goal of a qualitative study should be to have a large enough sample size to uncover a variety of opinions but to limit the sample size at the point of saturation. The final sample size was determined by thematic saturation, the point at which new data no longer appeared to contribute to the findings due to the repetition of themes and comments by participants. Additional focus groups were discontinued when it was determined that saturation was attained.

### Recruitment

For potential caregiver participants, the principal investigator (CC) worked with clinic staff and nurses to approach each potential participant at random in the clinic waiting room at each site, explained the nature of the study, and assessed the participant’s eligibility using a screening questionnaire. This generated a convenience sample of potential participants who were present in the clinic waiting area at each site on the days when the principal investigator (CC) was available to recruit for the study. Health promoters were recruited either by telephone or in-person at the clinics. All female health promoters were invited to participate. Those who expressed interest in participating were provided with the date, time, and location of the group discussion. As all participants might not be available on the day of the focus groups, several sources suggested over-recruitment by 20% to 50% [39,40], so goal recruitment was 9 participants per group.

### Data Collection

Data collection took place between December 2018 and February 2019. Before each FGD, informed consent procedures were administered and written informed consent was obtained, including consent to record FGDs with a digital recorder. Participant confidentiality was assured by using floral-themed names rather than their real names during the FGD so that participants could not be identified in the written transcripts.

Before starting the FGDs, we developed a semistructured FGD guide in English and then translated it into Spanish. Local stakeholders (ie, staff pediatricians, nurses, and other clinic staff) verified and revised the translation of preliminary interview guides. The development of the focus group guide was framed using the extended technology acceptance model (ETAM) [41] constructs, the information-motivation-behavioral skills (IMB) model [42] constructs, and scholarly literature, specifically from previous studies using focus groups as formative research for mHealth interventions in an LMIC [43] and in a high-risk population [44]. These semistructured discussion guides were iteratively refined after each focus group and modified to include new data gathered during discussions. The FGD guide for caregivers was designed to elicit which ICT they use, how they utilize ICT to access health information, what factors discourage and motivate caregivers to seek mHealth tools, and what features would be desired in a potential mHealth intervention. Caregiver groups were also asked to share their perspectives on influencing factors for EBF practices. The FGD guide for health promoters focused on identifying challenges with EBF and suggestions on how mHealth tools might be used to promote effective EBF practices.

FGDs were facilitated by a trained local research associate and were conducted in Spanish using the FGD guide. Training was provided by one of the senior researchers (LF) who has extensive experience in focus group facilitation. Before each session, a short survey was conducted to collect information on demographics, ICT use, and health information–seeking behaviors. Sessions lasted for 60-90 min and were audio-recorded. The principal investigator (CC) took field notes during each FGD. FGDs were conducted until thematic saturation was reached, as was determined when new information was no longer being obtained during discussions [45].

### Data Analysis

All digitally recorded interviews were transcribed verbatim in Spanish by a local transcriber. The transcripts were later professionally translated into English. Any private information accidentally revealed during the focus groups by a participant was removed. Transcripts were reviewed line by line by one of the authors (CC) to assess accuracy, perform framework indexing using the framework method [46], and start formulation of the codebook. A total of 2 authors (CC and SS), who are bilingual in English and Spanish, discussed 2 initial transcripts to refine the development of the codebook. Subsequently, all transcripts were carefully read by CC to develop a list of meaningful units corresponding to the major constructs of the mixed behavioral theory model, which were independently reviewed and discussed with SS. CC and SS then independently coded all transcripts using the established codebook. Coding discrepancies were discussed with LF and EL to obtain consensus. The data were coded using NVivo qualitative analysis software using directed content analysis following the theoretical framework. During coding, any additional meaningful units identified by coders were also identified and continuously discussed between the authors. Codes were arranged by meaning into major themes. Notable quotes pertaining to each theme were organized, discussed, and summarized in a document that presents the findings for each theme.

**Figure 1:**
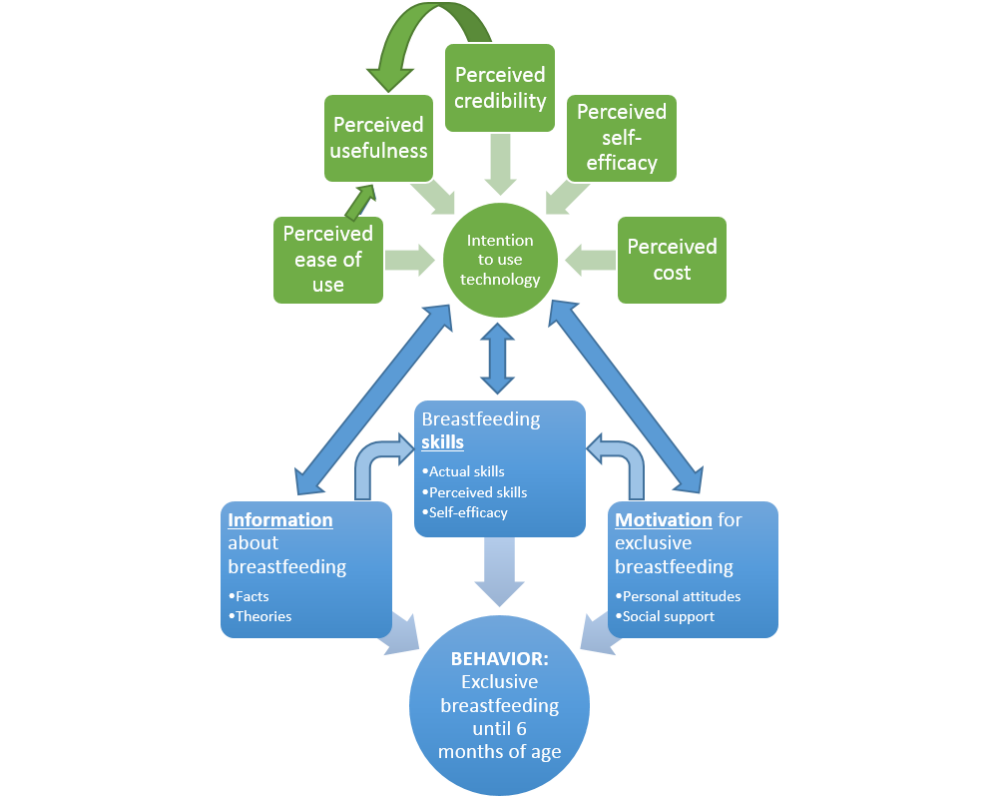
Combined conceptual model: extended technology acceptance model (ETAM) and information-motivation-behavioral skills model (IMB).

We chose a directed approach to content analysis through a combined deductive-inductive process to comprehensively review transcripts and identify salient themes [47]. Through deductive use of existing theory in a mixed model of the ETAM and the IMB model (Figure 1) [41,42], we were able to conceptually extend a theoretical framework to help determine the initial coding scheme and relationships between codes, while at the same time, adding any emerging themes from probing or freely shared topics by participants in an inductive approach [47]. In addition, several triangulation categories were used to enhance the reliability, objectivity, and validity of the results collected in this qualitative descriptive study: (1) data triangulation was achieved by administering focus groups with several participants at various times in Consuelo and La Romana, (2) investigator triangulation was achieved by correlating the findings from multiple researchers in the study to reach consensus, and (3) theory triangulation was achieved by using and correlating multiple theoretical strategies in the form of the mixed behavioral theory frameworks of the ETAM and the IMB model [45,47-49].

Applying relevant behavioral theories to an mHealth intervention is important because it can lead to well-developed strategies for health behavior change and health promotion [50,51]. They can also increase the effectiveness of digital tools and promote a receptive environment for their use [51]. For these reasons, we chose to ground our intervention with behavioral theories that addressed both our targeted behavior of EBF for up to 6 months and acceptance of digital technology. The IMB model was initially developed to promote HIV prevention interventions in inner-city minority settings [52]. The model supports the hypothesis that to initiate and maintain a desired behavior, adherence-related information and motivation must be provided, along with appropriate tools to maintain the behavior [42,52]. In previous research, the application of the IMB model has shown great promise in developing effective EBF promotion interventions in global settings [53,54]. A common model used to understand clinical staff and patients’ mHealth adoption is the technology acceptance model (TAM) [55]. Although the TAM has been a rigorously tested model in predicting user acceptance of an innovation, some have raised the need for the model to be extended and incorporated with further constructs to enhance its explanation and prediction of acceptance behavior [55,56]. Therefore, our study used an extended TAM that incorporates the TAM with the theory of planned behavior [41]. In addition to the original constructs of *perceived usefulness* and *perceived ease of use*, a trust-related construct (*perceived credibility*) and 2 resource-related constructs (*perceived self-efficacy* and *perceived cost*) are incorporated [41] to better predict caregiver and health promoter intention to use mobile phones for health information seeking.

## Results

### Overview

We conducted 6 FGDs consisting of (1) 7 health promoters from NPS, (2) 5 health promoters from CFLR, (3) 2 caregivers from MAMI, (4) 7 caregivers from CFLR, (5) 8 caregivers from MAMI, and (6) 6 caregivers from NPS. Demographics of the 35 participants are summarized in Table 1.

**Table 1:** Characteristics of focus group discussion participants.

Characteristics			Caregiver (n=23), n (%)		Health promoter (n=12), n (%)		Total (N=35), n (%)
Female participants			23 (66)		12 (34)		35 (100)
**Age (years)**							
	12-17	6 (26)		0 (0)		6 (17)	
	18-25	9 (39)		1 (8)		10 (29)	
	26-34	6 (26)		4 (33)		10 (29)	
	35-54	1 (4)		7 (58)		8 (23)	
	No answer	1 (4)		0 (0)		1 (3)	
**Clinical site of recruitment**							
	Niños Primeros en Salud, Consuelo, rural	6 (26)		7 (58)		13 (37)	
	Clínica de Familia La Romana, La Romana, urban	7 (30)		5 (42)		12 (34)	
	Módulo de Adolescentes Materno Infantil, La Romana, urban	10 (44)		0 (0)		10 (29)	
**Highest educational level achieved**							
	Completed middle school	9 (39)		1 (8)		10 (29)	
	Completed high school or technical trade school	7 (30)		6 (50)		13 (37)	
	Currently at university	3 (13)		4 (33)		7 (20)	
	Completed university	4 (17)		1 (8)		5 (14)	
**Employment status**							
	Unemployed	19 (83)		7 (58)		26 (74)	
	Employed^a^	4 (17)		5 (42)		9 (26)	

^a^Receives a salary for full-time work.

### Demographic Characteristics

All participants were female. Each of the 3 participating sites accounted for a similar number of participants, with approximately one-third of the total from each site. Thus, the majority were from La Romana (22/35, 63%). A total of 12 participants were health promoters in their respective community, and 23 participants were mothers of children aged ≤5 years currently receiving services at either NPS, CFLR, or MAMI. The major demographic differences between caregivers and health promoters were age, education level, and employment. Notably, caregivers were younger (15/23, 65% were aged <26 years), had less formal education, and had a higher percentage of unemployment (19/23, 83% vs 7/12, 58%) compared with health promoters.

### Participants’ Use of ICT and Sources of Health Information

On the basis of a brief survey conducted before each FGD, all participants (N=35) reported having a cellular phone, of whom 29 (83%) reported having a smartphone. Participants obtained health information from the internet, physicians, health clinics, family and friends, health promoters, and TV.

### Perceptions and Attitudes Toward mHealth

Most caregivers initially commented that they use ICT regularly to access health information. In the course of our FGDs, several factors influencing caregivers’ use of mobile phones regarding their child’s health emerged (Table 2). Prominent themes included ways caregivers use ICT, access limitations, perceived credibility of sources, perceived usefulness, cost, and privacy.

Caregivers utilized ICT in several ways. For example, some used ICT to access the internet to further research a diagnosis or medical terminology used by their doctor when they did not have enough time during their visit to ask. Some used mHealth to verify, confirm, or compare information received from different sources, including their family members and doctors. Others read or found health information using ICT and confirmed this information with a health professional to verify its validity or falsehood. Cumulatively, this health information–seeking behavior added further evidence to a major determining factor for caregivers’ use of a potential mHealth intervention: perceived credibility of source. Caregivers consistently expressed the importance of having information in the mHealth tool that was validated by credible sources, such as doctors, nurses, and other trained health professionals.

**Table 2:** Perceptions and attitudes toward mobile health from focus group discussions.

Model constructs		Example excerpts
**Perceptions and attitudes toward general health information**		
	Health information sources	“I have a smartphone by which I can research on Google and YouTube about medication, about how to use them, about breastfeeding too. I inform myself by this form of communication: a smartphone.” (NPS^a^ health promoter, rural setting) “There is a webpage that I used when I was pregnant. And there is another page that I follow, called: ‘Lactating Mothers’. It informs you about the development of your baby, growing up, and all of the different things that happen.” (MAMI^b^ mother, urban setting)
	Health information source considered most trustworthy	“Directly with doctors, because you can find so many things on the internet and you don’t know which ones are real. I directly call the doctor or I go to clinic.” (CFLR^c^ mother, urban setting)
	Health information–seeking behavior	“Sometimes the doctor can give a diagnosis or use medical terminology that we don’t understand very well and, because of time, we don’t ask the doctor. But we search on the internet. We search and we get all the information in layman’s terms. The internet is really helpful.” (CFLR health promoter, urban setting) “Apart from the doctor, for your own knowledge, it’s important to know what other sources say and also to search information on your own. To investigate and to have the opportunity to look for information and to learn.” (NPS mother, rural setting) “People that lived through the process like my mother and my mother-in-law. People who have previous knowledge about motherhood. And for something rare, I call the pediatrician.” (CFLR mother, urban setting)
**Facilitators and barriers to mobile health use for child health**		
	Perceived credibility of source	“Sometimes we get confused, because a person comes and says something and then another one comes with another explanation. So it's better to look for people like promoters, psychologists, or doctors... It's better to ask them. They are trained people. Because sometimes people upload things and share information, but you get confused; then you ask yourself: Is it true or not?” (NPS health promoter, rural setting) “It’s not so bad. It has its pros and cons. What we have to do when we get information on the internet is to confirm it with the pediatrician. There is a lot of good information on the internet and many times we build on what we know and gain knowledge. And it’s important.” (CFLR mother, urban setting)
	Perceived usefulness	“Creating this app is a good way to give orientation about breastfeeding. Every mother can have it on her cellphone, because almost all mothers put more attention to their phone than to talks. Having the information on their phone, in order to have everything there, is a good idea. So they have the app and have access to the information.” (NPS health promoter, rural setting) “For me it’s quite useful, because many people don’t constantly remember things. Having a reminder of everything: vaccines, medicines, is very important. Because there are vaccines that if you don’t get them in time, can cause harm to the child.” (MAMI mother, adolescent, urban setting)
	Perceived ease of use	“I feel very comfortable, because it offers you the information instantly. When you want to know something and there is no one nearby you can ask; for example if the pediatrician is occupied or the doctor is receiving another patient and he cannot answer you in that moment. Then you have the information there, immediately.” (CFLR mother, urban setting)
	Barriers to ease of use	“There can be problems with it. Because there are people that can’t use technology well. There is good information, but some people don’t know how to use it.” (NPS mother, rural setting)
	Perceived self-efficacy	“[I feel] very comfortable because [technology] does not have time limits. I feel that I’m not bothering anybody. And I can easily dedicate time to it and I can easily find what I’m looking for.” (CFLR mother, urban setting)
	Perceived cost	“Well, it’s not so easy, because to obtain a mobile phone you have to pay monthly and it’s not cheap. And you don’t have money to pay every month. Sometimes they cut off the service and you cannot communicate; nothing. You can make calls but without internet you can’t do anything.” (NPS mother, rural setting)
	Perceived loss of privacy	“I think I would not [use an application asking for private information]. But I have Facebook and Facebook asks for your name and telephone number.” (NPS mother, rural setting)

^a^NPS: Niños Primeros en Salud.

^b^MAMI: Módulo de Adolescentes Materno Infantil.

^c^CFLR: Clínica de Familia La Romana.

Perceived usefulness was another influencing factor for mHealth use, which was described among participants. Some caregivers described the usefulness of an mHealth tool both in terms of what they had encountered and what they wish were available. Desirable characteristics and capabilities include quick and ready access to information, the ability to send reminders for important necessities such as vaccines and medications, and helpfulness with decision-making regarding their child’s health. Several health promoters also explicitly mentioned how an mHealth tool would be especially useful to them during their home visits and provide them with an educational resource to share with their clients and families. Other factors identified among caregivers include perceived ease of use and perceived self-efficacy.

Some barriers to mHealth use among caregivers included perceived cost and perceived loss of privacy related to use. For example, many caregivers mentioned the difficulty of maintaining monthly internet service due to cost. If there were an additional cost of the mHealth tool, most mentioned they would not pay for it. However, some argued that if the mHealth tool seemed to add value to their lives, they would pay for it. When asked how they felt about sharing private information through the mHealth intervention, many expressed concerns about privacy and indicated that they would potentially input false personal information into a nonsecure ICT to maintain their privacy.

### Perceptions and Attitudes Toward Breastfeeding and Influencing Factors

Participants observed and shared various factors that influenced their breastfeeding practices, which could be relevant to the development of an mHealth EBF promotion tool (Table 3). Most participants were aware of the benefits of breastfeeding for both their children and themselves, and some participants mentioned additional benefits such as economic savings and environmental benefits (eg, less waste production and reduced water consumption with no need for bottles and formula). Most participants were aware of the recommended time to initiate breastfeeding and the recommended duration of EBF. Change in knowledge was mentioned across the different focus groups as a major factor important to promoting breastfeeding. Participants proposed that this could be achieved through support or information from experienced family members or health care workers and through dispelling myths shared in the community.

Despite their knowledge of the benefits of EBF and intentions to exclusively breastfeed, detailed probing revealed that supplementing with water or formula in the first 6 months of life is common. Mothers provided reasons that included urging from the infant’s grandmother to supplement, returning back to work, feelings that the baby was still hungry or that breastmilk production was inadequate, and previous experience with another child. Several mothers from the adolescent clinic (MAMI) also mentioned that their infant’s grandmothers offered their babies beans, coffee, and other foods besides breastmilk as the initial food when their child was born.

Many mothers described their specific memories about breastfeeding, some reporting their difficulties and others sharing their positive experiences. Some shared challenges related to breastmilk production, physical pain, newborn refusal to latch, and preference to give formula. Other mothers, who shared positive experiences with breastfeeding, mentioned a feeling of having a stronger bond with their infant, health benefits to their infant (such as falling ill less frequently or seeing their rapid growth), and observing benefits for themselves (such as relief from breast engorgement or feeling thinner).

**Table 3:** Perceptions and attitudes toward breastfeeding from focus group discussions.

Model constructs			Example excerpts
Initial feeding practices			“Yes, my mom gave him beans.” “My mom gave coffee to him.” “Enfamil^a^, because I couldn’t endure the pain of my breasts. They hurt a lot.” (MAMI^b^ mothers, adolescents, urban clinic) “When my son was born, I immediately placed him on the breast and I continue to breastfeed him.” (NPS^c^ health promoter, rural clinic) “At first I breastfed her, but she didn’t like it. It seemed it was salty and I didn’t insist. I bought her formula.” (CFLR^d^ health promoter, urban clinic) “Well I practiced exclusive breastfeeding with my baby. Sometimes a little water, until 6 months, when I began to work.” (CFLR mother, urban setting)
**Facilitators and barriers to breastfeeding**			
	**Information about breastfeeding**		
		Benefits of breastfeeding	“Breastfeeding is very good, because the baby receives all the nutrients. But it’s also very healthy for the baby and the mother, because doctors say that breastfeeding mothers have less probability to develop cancer. Babies grow up healthy, they don’t get sick as often and it’s very important.” (CFLR health promoter, urban setting) “Breastfeeding has a lot of benefits. The child socializes with their mother. You save money. You don’t have to bring anything, because the mother is already carrying her child’s food. It is good for the environment. I have a benefit, the child has a benefit, the environment has a benefit. Everybody benefits.” (NPS health promoter, rural setting)
		Myths surrounding breastfeeding	“...There are breastfeeding mothers who are giving water apart from breastmilk, to the baby... It’s important to explain to them...the baby doesn’t need additional water, because it’s in the milk.” (CFLR health promoter, urban setting) “I think that people believe one of the biggest inconveniences [of breastfeeding] is the myth that they will get very skinny and that the breasts are going to sag.” (NPS health promoter, rural setting)
		Change in knowledge	“Well, for me, there is the challenge with my next child to practice exclusive breastfeeding, because my mom always told me to give breastmilk in addition to the formula, but nobody ever told me to breastfeed exclusively.” (CFLR mother, urban setting)
	**Motivation to breastfeed**		
		Personal attitudes	“Breastfeeding suits me and it suits the child. It’s convenient for me, because while breastfeeding he becomes better acquainted with me. If he feels fussy or anxious, I begin to breastfeed him and he immediately calms down, because he knows that I stay with him. Because, since the child was in the mother's womb, his best friend is the mother's heart.” (NPS health promoter, rural setting) “It’s also very good for us, women, because we get skinny (laugh). For this reason I breastfeed. You believe that it’s because I like to give it, but no. It’s to get skinny. Look at this belly!” (MAMI mother, adolescent, urban setting)
		Support received	“As I was a first time mother, my baby’s grandmother urged me, because I didn’t want [to breastfeed]. I was engorged. She said ‘Give her breast!’ and the milk finally letdown. She told me ‘Give her breast! It helps her grow.’ And then, with the second one, nobody had to tell me anything, because I already knew, because of what I had experienced with the first one.” (NPS mother, rural setting)
		Support desired	“There are partners who believe that taking care of the baby is the obligation just of the mother. So there are fathers not supporting the mother in taking care of the baby. And I think it’s work of 50% father and 50% mother.” (CFLR health promoter, urban setting) “It would be a good, an excellent idea [to have an application for breastfeeding], because right now, there are a lot of pregnant teenagers and they have no information about breastfeeding, about the consequences of breastfeeding or not breastfeeding.” (NPS health promoter, rural setting)
	**Skills and experience with breastfeeding**		
		Perceived self-efficacy to breastfeed	“At first, I felt a lot of pain; and then I got used to it and the nipples let down... I spoke with her and I carried her. She was tiny and I spoke with her and I got used to it. I loved giving my baby breast at a walking pace.” (MAMI mother, adolescent, urban)
		Successful breastfeeding experience	“I had a very pleasant experience. Because I had to breastfeed twins, I will never forget. It was something very new for me, because breastfeeding two children at the same time is a bit difficult, but I learned that breastfeeding is important, because it helps our children’s nutrition and that the development of our children depends on it, as they grow. Because if we breastfeed, they will have the antibodies they need for any disease. I learned that it is important to breastfeed.” (NPS health promoter, rural setting) “Breastfeeding the baby is very good. I breastfed mine for 1 year and 10 months. And this baby never got sick, thank God.” (CFLR mother, urban setting)
		Unsuccessful breastfeeding experience	“My experiences [with breastfeeding] were not very long. I have two children. A boy that is 7 years old and a girl that is 6 months. The boy breastfed until he was 3 months. I tried to put him on the bottle [with expressed breastmilk], but he didn’t want it anymore and me neither. The girl wanted to stop after 2 months. I pumped breastmilk and I gave it to her, but she didn’t want it anymore.” (NPS mother, rural setting) “When I saw the blood coming out of my breasts, I was scared. My nipple was cut by so much breastfeeding. I had cuts and I said: ‘Oh my God! I have to stop it! I have to save myself from that!’” (CFLR health promoter, urban setting)

^a^Brand of infant formula.

^b^MAMI: Módulo de Adolescentes Materno Infantil.

^c^NPS: Niños Primeros en Salud.

^d^CFLR: Clínica de Familia La Romana.

### Desired Features for a Potential mHealth Intervention to Address EBF

In addition to sharing their perceptions and attitudes toward mHealth in general, participants provided their recommendations for important features to include in a potential mHealth intervention to promote EBF (Table 4).

A frequently mentioned recommendation on how to introduce mHealth for EBF support and promotion was having the ICT cover other topics related to their child’s health in addition to breastfeeding, such as growth and development, vaccines, prenatal and postnatal health, and common ailments. Other factors perceived to be important included (1) using an app that would be easily accessible on a smartphone with remote access due to commonly inconsistent internet service; (2) having an interactive component, whether through video call or face-to-face consultation; and (3) using simple, educational, and motivational messaging with video and images. Others suggested using *myth-busting* messages. Health promoters and pregnant women were also identified as specific groups that could especially benefit from this mHealth tool. For example, health promoters suggested having a platform to be used as an educational tool for home visits. Participants also proposed targeting pregnant women who could have access to this information while preparing to give birth and raise a child.

**Table 4:** Desired features of a potential mobile health intervention: major themes from focus group discussions.

Major themes		Example excerpts	
Message content based on specific topics		“To speak about hygiene, about fever, about the most common diseases, about infections he can have, about influenza which is very common, and growth.” “I would like information about nutrition and home remedies for children.” (MAMI^a^ mothers, adolescents, urban setting) “A reminder for vaccines and, if possible, the information about which vaccines the baby should get would be very important.” “I’m not sure it’s good to fill the application with too much information. But maybe you can put information about pregnancy... It would be very useful.” (CFLR^b^ health promoters, urban setting)	
Timing of information		“For me, it’s important to get it daily, not flooding people with messages, but with one paragraph specifying something on the theme. In the notification, you want to know about this topic, or in a video, specifying all you have to do. For example, if they speak about how to feed the baby after [6 months of] breastfeeding. It’s important to specify, more or less, the food that you can give to him, how and the quantity.” (CFLR mother, urban setting)	
Preferred method of delivering information		“An application would be good because I can have it on the phone, so whenever I need information, I go to the application directly. I suppose the application will contain the videos and the texts that people are looking for. So, I prefer an application.” (NPS^c^ health promoter, rural setting) “I also like face-to-face. I like it, because when you speak with the other person, you can see how they receive the message, and you can say: ‘Did you understand? Repeat it!’ You can understand better face-to-face. Videos too. Through videos you can live what you are seeing. So, I like both, but mainly face-to-face.” (CFLR health promoter, urban setting)	
Message intent		“Motivational. ‘Care for your baby like you care for yourself!’” (MAMI mother, adolescent, urban setting) “I recommend informing about breastfeeding, because there are many myths that one believes, because I have mine too. The pediatrician, health staff, application or a webpage can dispel these myths in order to give better benefits to children.” (CFLR mother, urban setting)	
Target audience or target strategy for an eHealth tool		“[An] application is very good, because we are [health] promoters. We go to a mother’s house to do a home visit and through the app we can show her the information. A lot of them don’t pay much attention to what we say sometimes. So I can show them that through the app, she can find all the information about vaccines, breastfeeding… and I can show it to my family and to the entire community.” (NPS health promoter, rural setting) “In this app, I want to find themes related to breastfeeding; about pregnancy, how to be prepared; because a mother can access it and she can find everything related to her and to her baby... So that when a mother gives birth, she knows how to nurse him, she knows the benefits of breastfeeding and she knows what happens with formula. Sometimes we can say to a mother that breastfeeding is the best, but sometimes they don’t trust us. But if they have information, they can say: ‘Wow, it’s true. Look! Here it tells me!’ Sometimes they want evidence. If we have this app, it’s much better.” (NPS health promoter, rural setting)	
**Other features**			
	Telemedicine	“If you are at home and the baby has a problem, you can start a video call and you can ask at that moment. [‘The trained professional’] can tell you what to do, before taking the baby to the doctor.” (NPS mother, rural setting)	
	Remote access	“Because it may be that I won’t have internet on my phone and that I can’t search something on the application. If I don’t have money to activate service, it would be good if it does not need internet connection.” (NPS mother, rural setting)	
	Myth busting	“It would be good to scientifically define the origins of the myths and discredit them or not.” (CFLR health promoter, urban setting)	

^a^MAMI: Módulo de Adolescentes Materno Infantil.

^b^CFLR: Clínica de Familia La Romana.

^c^NPS: Niños Primeros en Salud.

## Discussion

### Principal Findings

Mobile phones are increasingly common in the Dominican Republic, where, according to the World Bank, there were 84 mobile cellular phone subscriptions per 100 people in 2018 [57]. In addition, smartphone use in this setting is increasing rapidly; a 2019 survey found that 61% of the Dominican population owned a smartphone compared with 51% in 2017 [58]. This rapid expansion of access to mobile technology creates an opportunity to develop health-related interventions to meet the needs of rural and urban communities across the Dominican Republic. This study provides novel insights into community and caregiver perspectives of a potential mHealth intervention to promote EBF for women with children receiving care at multiple clinical sites in the Dominican Republic. This study also highlights numerous aspects of the content and service delivery model that may affect users’ acceptance and impact of the intervention. By identifying caregiver and health promoter opinions on a potential mHealth tool using behavior theory models, the results can inform effective future mHealth intervention design.

Effective mHealth research should aim to provide a richer understanding about the nature of the cultural factors [59] that shape the adoption and success of these new technologies. This study aimed to address the gap in mHealth research related to user acceptability and the development of theory-based interventions. There is a need for more mHealth interventions grounded in behavior change theory [28,59] that explore the psychological, cognitive, and behavioral dimensions of maternal and child health, which served as the foundation of our design.

We do not have similar previous studies for comparisons, as this study is, to our knowledge, the first study of its kind in the Dominican Republic. However, other studies have assessed the feasibility and acceptability of mHealth apps aimed at improving breastfeeding in other countries [27-30]. For example, in Saudi Arabia, more mothers expressed their intention to practice EBF after receiving mHealth-based education about EBF and early breastfeeding initiation [27]. An Australian study reported high ratings for an evidence-based breastfeeding app designed to provide men with social support and information to enhance the help they can offer their breastfeeding partners [28]. A Thai study demonstrated the potential for an mHealth app to be a useful self-management tool for breastfeeding mothers [29]. In the United States, researchers at the University of Missouri found that stakeholder and user engagement indicated that mHealth has the potential to be a useful strategy for providing breastfeeding support to mothers [30]. These studies, combined with the findings of our study, indicate the potential positive impact an effectively designed stakeholder-informed mHealth tool can have in promoting EBF.

Mobile phones provide an opportunity to improve health behaviors, as evidenced by caregivers in this study who mentioned that their intention to breastfeed may have been enhanced if they had better access to trusted information about EBF from an mHealth app. According to the 2011 World Health Organization report on mHealth, mobile phones provide a new communication channel for health promotion and community mobilization [60]. Multiple studies have shown promising results of using mobile phones and text messaging to improve nutrition [27-30,61,62]. Multiple systematic reviews have evaluated the impact of mHealth interventions on maternal and child health in LMICs [59,63-67]. These reviews have noted that although a handful of interventions have shown some promise in improving health outcomes compared with routine care, most studies lack high methodological quality, such as theory-based design, standardization of content, and validated outcome measurements [59,63-67]. This observation is important not only during the mHealth design phase but also in the future evaluation of the resulting mHealth intervention.

### Applying Findings to Future Intervention Design

Our findings provide an additional context to the low EBF rate in this population that can inform future breastfeeding interventions. On the basis of participant responses with probing, mixed feeding (a combination of breastmilk and formula) appears to be common. The reasons given for this practice, such as returning back to work, feeling the baby is still hungry, and feeling like breastmilk production is inadequate, have also been mentioned in previous studies [68-70]. Thus, this work confirms that it will be important to address factors associated with mixed feeding in a future mHealth intervention aimed toward this community and others with similar practices.

Our findings suggest that caregivers and health promoters in this setting prefer to utilize a low- to no-cost health app that is easily accessible on mobile phones, regardless of internet service. This supports previous research examining trends in mHealth in LMICs [71]. Participants also generally preferred to have access to a trustworthy trained individual or specialist to navigate and promote EBF. In this setting, it is culturally accepted, and many times sought after, to have face-to-face interactions with trained professionals to obtain health education and consultation. As suggested by participants, this desired component can be applied to an mHealth intervention by either adding a video call feature or discussion forum component to the intervention. Participants also suggested that during health visits, physicians, nurses, or health promoters can use the mHealth tool to explain standardized health information and provide access to the mHealth tool for caregivers to use beyond the encounter. Similar findings have been reflected in previous research in the development and assessment of mHealth interventions in other countries, including India and Germany [12,72,73].

Perceived loss of privacy is an important barrier to mHealth use mentioned by participants, which has also been described in other studies [74,75]. As illustrated in the quotes in Table 2, there are conflicting attitudes toward providing private information in mobile apps. Although some participants mentioned that they would not use an app that required private information, some reported that they would input false personal data to access the app. Regardless of privacy concerns, popular apps such as Facebook were still used among our participants, which may indicate that functionality and convenience may take precedence over privacy concerns. Further research is needed to address concerns regarding privacy and security in future mHealth apps and to explore if it is necessary to be specifically customized for different purposes or users.

Caregivers in our study expressed that although an mHealth tool to promote EBF would be useful, it would be desirable if the tool could provide additional information and self-management support for other maternal and child health topics, such as growth and development, vaccines, prenatal and postnatal health, and common childhood illnesses. This corresponds with previous literature in which caregivers described mobile apps to be better suited for more broad topics that would be accessed more frequently or to meet a repeated, unique need to be worth the significant space and data on their devices [76]. In addition, in considering the design of an impactful mHealth tool that is highly valued and frequently used by parents, a previous systematic review recommended combining educational elements with troubleshooting support [71]. Future intervention designers should take these considerations into account to provide optimum benefit for the intended target population.

### Strengths

A strength of this study is inherent in its design and employment of several categories of triangulation (data, investigator, and theory) to enhance the reliability, objectivity, and validity of the results collected [45,47-49]. In particular, data triangulation allowed for the collection of different perspectives from female caregivers and health promoters of various ages living in both rural and urban settings in the Dominican Republic. There are several examples that illustrate triangulation in our data. As seen in Table 3, there is an overlap between a myth about breastfeeding and motivation for breastfeeding. A rural health promoter mentions that “one of the biggest inconveniences [of breastfeeding] is the myth that [mothers] will get very skinny,” whereas later, an urban caregiver describes how getting skinny was a motivation for her to breastfeed. The second example of triangulation appears when a rural health promoter mentions that she would like support aimed toward pregnant teenagers because they have “no information about breastfeeding” (Table 3). However, an urban teenage mother mentions a specific website called *Lactating Mothers*, which she uses to seek health information regarding her child and breastfeeding (Table 2). These findings form a strong evidence base and source of various perspectives from which mHealth interventions that seek to improve EBF in the Dominican Republic and other similar settings can be designed.

Given the extensive description of the methodology and design of our study, researchers interested in conducting similar work can better understand how our findings might apply or relate to their target population [77]. This study integrates the perspectives of stakeholders (caregivers and health promoters) throughout the design process. Formative research with stakeholder involvement is foundational to delivering effective maternal and child health educational interventions and facilitating a more sustainable and broader dissemination [76]. Furthermore, within the global health community, we must look across specific content areas toward the broader themes emerging within the literature on technology-based interventions. Consistent with previous studies, this serves as a call to action to pair formative research with strong evidence-based design, combining messaging type and content with optimum technology platforms to effectively improve health outcomes [17,18,28,59,63-67,78].

### Limitations

Despite these strengths, there are some limitations that should be considered. There is an inherent selection bias owing to our recruitment strategy, which involved approaching potential participants in the clinical site waiting areas. One might argue that women living in local communities, who are not actively seeking primary care services, may have the greatest need for breastfeeding education and guidance. This selection bias might have been avoided if we had sought help from the health promoters to identify women in the community who do not use primary care services. However, as free services are highly accepted, women not accessing these services are less representative of the population as a whole. Another limitation is that the FGD guide was not modified to elicit the unique perspectives of mothers in the older age groups or to address any contradictions seen in the data. There was a missed opportunity to further identify barriers to EBF, such as exploring reasons for mixed feeding practices among older mothers, or provide a direct context to the contradictions observed. Third, it must be mentioned that the principal investigator was also a staff pediatrician at 2 of the clinical sites (NPS and CFLR). Although she did not facilitate the FGD, she was present for notetaking and observing the groups in each session. Owing to this, participants may have modified some aspects of their responses toward breastfeeding with the awareness of being observed (Hawthorne effect) [79]. However, the fact that participants freely admitted to behaviors such as mixed feeding, which they likely knew to be contrary to the pediatricians’ recommendations, suggested that they felt free to express their true behaviors and feelings in the presence of a pediatrician.

Our study findings may not be generalizable to communities in the Dominican Republic where levels of employment are higher. On the basis of data from the World Bank, in 2019, the labor force participation rates (eg, percentage of people aged over 15 years who are employed or actively looking for work) were 51% female and 77% male [80], compared with 26% of our all-female study cohort. A unique distinction about the clinical sites in this study is that medical services are significantly subsidized for patients. For example, patients who qualify for NPS services have no fees for visits, laboratories, or medications. To be a patient at NPS, children must be aged ≤5 years and live in 1 of the 8 *barrios* (neighborhoods) served, which are the poorest in Consuelo. In addition, at CFLR, people living with HIV receive government-funded highly active antiretroviral therapy (HAART) free of charge. Laboratories and medications are offered at a significantly lower price than at other facilities in La Romana. This provides context as to how recruited participants are able to receive care despite high unemployment rates.

### Conclusions

There is a high level of acceptance for ICT tools, particularly mHealth apps, for the promotion of breastfeeding and child health among caregivers in this setting. mHealth tools can contribute to increased breastfeeding self-efficacy, and hence, identifying the desirable features of such tools will create impactful interventions in both rural and urban settings in the Dominican Republic. Future mHealth interventions should be designed using formative research with stakeholder involvement. Ideally, the mHealth tool should implement the following features:

- minimize barriers to use, such as low cost and access without active internet service
- promote frequency of use by adding perceived value to caregivers, such as providing both educational and self-management content
- address the cultural needs and acceptability of users based on behavioral theory models.

In general, caregivers want to receive trusted and reliable information that is easily accessible through various formats, and they are interested in information beyond breastfeeding.

## Abbreviations

CFLR: Clínica de Familia La Romana
EBF: exclusive breastfeeding
ETAM: extended technology acceptance model
FGD: focus group discussion
ICT: information and communications technology
IMB: information-motivation-behavioral skills
LMICs: low- and middle-income countries
MAMI: Módulo de Adolescentes Materno Infantil
mHealth: mobile health
NPS: Niños Primeros en Salud
TAM: technology acceptance model
